# Surgical outcomes following laparoscopic major hepatectomy for various liver diseases

**DOI:** 10.1097/MD.0000000000005182

**Published:** 2016-10-28

**Authors:** Sung-Hwa Kang, Ki-Hun Kim, Min-Ho Shin, Young-In Yoon, Wan-Jun Kim, Dong-Hwan Jung, Gil-Chun Park, Tae-Yong Ha, Sung-Gyu Lee

**Affiliations:** a Department of Surgery, Dong-A University Medical Center, Dong-A University College of Medicine, Busan; b Division of Hepatobiliary Surgery and Liver Transplantation, Department of Surgery, Asan Medical Center, University of Ulsan College of Medicine, Seoul, Korea.

**Keywords:** Laparoscopic liver resection, major hepatectomy

## Abstract

The aim of the study was to report surgical outcomes (efficacy and safety) of laparoscopic major hepatectomy for various liver diseases.

Although the number of laparoscopic liver resections has increased, expansion of laparoscopic major hepatic resection remains limited, mainly owing to the technical difficulties for the procedure as compared to open surgery. We describe our experiences with laparoscopic major hepatectomy for various liver diseases.

We retrospectively reviewed the medical records of 192 patients who underwent laparoscopic major hepatectomy between October 2007 and March 2015 at Asan Medical Center, Korea.

The mean age of the patients was 54 ± 11.6 years, and their mean body mass index was 23.5 kg/m^2^. The most common preoperative diagnosis was hepatocellular carcinoma (n = 82, 42.7%), followed by intrahepatic duct stones (n = 51, 26.6%). We performed 108 left hepatectomies, 55 right hepatectomies, 18 right posterior sectionectomies, 6 right anterior sectionectomies, 2 central bisectionectomies, and 3 donor right hepatectomies. The conversion rate was 1.6% (3 cases) due to bleeding, bile leakage, and uncontrolled hypercapnea during the operation. The mean operation time was 272 ± 80.2 minutes, and the mean estimated blood loss was 300.4 ± 252.2 mL. The mean postoperative hospital stay was 9.8 days. All resection margins were tumor-free in cases of malignant tumors. The morbidity rate was 3.1% (n = 6), including for case of biliary stricture. There were no deaths.

Laparoscopic major hepatectomy, including donor hepatectomy, is a safe and feasible option for various liver diseases when careful selection criteria are used by a surgeon experienced with the relevant surgical techniques.

## 1 Introduction

Laparoscopic hepatectomy was first introduced in the early 1990s.^[^1 2^]^ Since then, it has rapidly evolved into a safe and feasible option for treatment of various benign and malignant liver lesions. The technique has shown low postoperative complication rates and good oncological outcomes without increasing intraoperative complications. Improvements in biomedical technology and accumulation of surgical experience have led to a global increase in the number of laparoscopic hepatectomies performed.^[^3–10^]^

Initially, laparoscopic hepatectomy was confined to minor liver resections, such as left lateral sectionectomy, and the patient selection criteria were strict. Despite the clear feasibility and safety of laparoscopic minor hepatectomy, laparoscopic major resection remains challenging due to technical difficulties and fear of uncontrolled bleeding, as in open surgery. Nevertheless, available data support laparoscopic major hepatectomy as being a safe and efficient procedure when performed by an experienced surgeon on selected patients.^[^11–17^]^ We have >20 years of accumulated experience with open liver surgery and laparoscopic surgery for various liver resections with a high success rate, and have accumulated so far 10 years experience with laparoscopic major hepatectomies. Herein, we present our experience with laparoscopic major hepatectomy for various liver diseases in terms of outcomes of surgeries performed over the past 8 years. We document our surgical technique and discuss the feasibility and pitfalls of laparoscopic major hepatectomy.

## 2 Methods

After approval from our Institutional Review Board (AMC IRB 2015–1399), we retrospectively reviewed the medical records of 192 patients who underwent laparoscopic major hepatectomy from October 2007 to March 2015 at Asan Medical Center, Seoul, Korea. All operations were performed by a single surgeon (KH Kim). We included only pure laparoscopic anatomical major resections, excluding hand-assisted and laparoscopy-assisted methods. In open liver surgery, the definition of major hepatectomy was a resection of 3 or more Couinaud segments. The definition of major hepatectomy in laparoscopic liver surgery was expanded to resection including postero-superior segments 4a, 7, and 8 as they are difficult to access.^[18]^ In this study, central bisectionectomy, right anterior sectionectomy, and posterior sectionectomy were classified as major hepatectomy.

### 2.1 Patient selection

All patients had good preoperative performance status (American Society of Anesthesiology classes I to III). Routine blood tests, measurement of viral titers, indocyanine green retention rate at 15 minutes (ICG R15), triphasic liver dynamic computed tomography (CT), and double contrast magnetic resonance imaging (MRI) were performed in all patients. Additionally, positron emission tomography (PET) scans, chest CT scans, and tumor markers were assessed in patients who had malignant liver diseases. Patients who had significant extrahepatic disease and Child-Pugh classification B or C cirrhosis were excluded. We checked the expected remnant liver volume for all patients by CT liver volumetry. The cut-off value was 35% of total liver volume for all patients. Tumors that were bilateral, large (>10 cm), involved major hepatic or portal veins, or were located within 1 cm of the hepatic hilum or inferior vena cava (IVC) were excluded for laparoscopic major hepatectomy. In donor right hepatectomies, we used very strict selection criteria for 3 donors: a single right hepatic artery, normal portal vein and bile duct anatomy, and no inferior hepatic vein that would require reconstruction.

### 2.2 Surgical technique

**Figure 1:**
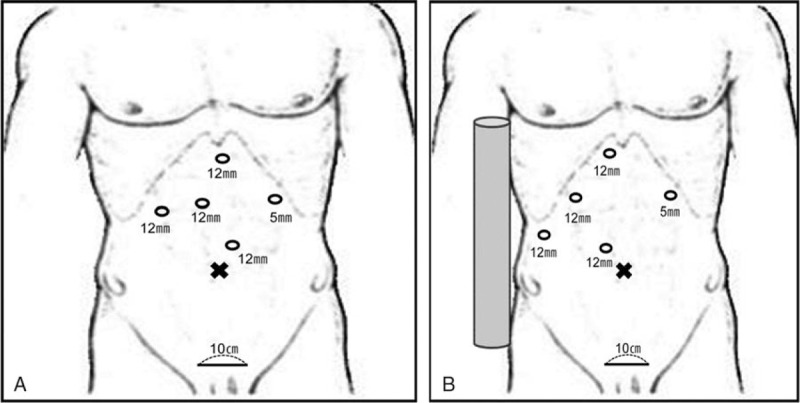
(A) Position of trocar for left hemihepatectomy, (B) position of trocar for resection of right side liver such as right hemihepatectomy, right anterior and posterior sectionectomy and central bisectionectomy.

The patient was placed in the supine and mild reverse Trendelenburg position, with legs spread apart. A pillow was usually placed under the right side of the patient's back in cases of right side resection. The operating surgeon stood between the patient's legs. The assistant surgeon and the scopist were positioned on the patient's left. An intermittent pneumatic compressor was used on the legs of all patients to prevent deep vein thrombosis. Five trocars were used in all procedures: four 12 mm ports and one 5 mm port. Trocar position was determined by the type of operation and the location of the tumor (Fig. 1). We used a 10 mm, 30 degree camera. Pneumoperitoneum was established with a Veress needle and the intra-abdominal pressure was maintained at 12 mm Hg with a dual CO_2_ gas supply channel.

After dividing the round ligament, dissection of the falciform ligament was performed in the cephalad direction and the root of the hepatic vein was exposed. For right side resection, such as right hemihepatectomy, right anterior and posterior sectionectomy and central bisectionectomy, inflow was controlled by the Glissonean approach. After cholecystectomy, the peritoneum of the hepatoduodenal ligament was dissected at the hilar region. The glissonean pedicle was encircled extraparenchymally using a Goldfinger dissector (Ethicon Endo Surgery, Johnson & Johnson, New Brunswick, NJ). The isolated Glissonean pedicle was clamped with a laparoscopic bulldog clamp, and then the demarcation line was identified and marked with electrocautery. For left hemihepatectomy, cholecystectomy was not performed for traction during parenchymal transection, unless there was no pathologic finding in the gallbladder. To control the inflow, the left hepatic artery and left portal vein were isolated and ligated individually. The left bile duct was divided intraparenchymally using a vascular stapler after parenchymal transection.^[19]^

Before parenchymal transection, assessment of tumor locations and vascular structure was performed using intraoperative ultrasonography with a flexible laparoscopic probe. The Pringle maneuver had not been used prior to laparoscopic major hepatectomy because of the fear of liver ischemia and lack of appropriate instruments. After accumulation of surgical experience and application of the laparoscopic bulldog clamp, we began to use the Pringle maneuver to prevent bleeding during parenchymal transection. The superficial parenchyma of the liver was transected using energy devices such as Enseal, Harmonic scalpel (Ethicon Endo-Surgery Inc., Cincinnati, OH) and Thunderbeat (Olympus Medical Systems Corp., Tokyo, Japan). Deep parenchymal transection was accomplished with a combination of a Cavitron Ultrasonic Surgical Aspirator (CUSA; Excel, Integra Lifesciences Co., Plainsboro, NJ) and the energy devices. Small branches of the hepatic vein were ligated with an endoclip. The hepatic vein, bile duct, and Glissonean pedicle were transected using a vascular stapler. For donor right hepatectomies, the right hepatic artery and right portal vein were identified and individually isolated using an atraumatic grasper (Direct Drive Laparoscopic Grasper; Applied Medical Resources, Rancho Santa Margarita, CA) and a monopolar dissector. The Pringle maneuver was not used during parenchymal transection in order to prevent graft damage. The right hepatic duct was exposed after complete transection of the hepatic parenchyma and was cut just above the Hem-O-Lock clip (Weck Closure System, Research Triangle Park, NC), which was clipped onto the proposed target level of the right hepatic duct following intraoperative cholangiogram by the C-arm. Contrast medium was infused into the cystic duct through a Cobra tube (Torcon NB^®^ Advantage Catheter, Cook Inc., City, IN). A 30-mm Endo TA unilateral linear stapler (US Surgical, Norwalk, CT) was used to cut the right hepatic vein.

The resected specimen was retrieved using a plastic bag via a suprapubic incision. After hemostasis and irrigation of the surgical bed, fibrin glue was used on the resected liver surface and 1 closed-suction drainage tube was placed near the surgical bed.

### 2.3 Statistical analysis

Categorical variables are summarized using proportions and continuous variables are presented as means with standard deviations. Disease-free survival and overall survival rates were calculated using the Kaplan–Meier method. All analyses were performed using IBM SPSS Ver. 20.0 (IBM Co., Armonk, NY).

## 3 Results

A total of 192 patients (93 males, 99 females) underwent laparoscopic major hepatectomy including 108 left hemihepatectomies, 55 right hemihepatectomies, 18 right posterior sectionectomies, 6 right anterior sectionectomies, 2 central bisectionectomies, and 3 donor right hemihepatectomies (Table 1). The mean age of the patients was 54.2 ± 11.6 years and their mean body mass index was 23.5 kg/m^2^. American Society of Anesthesiology classes I, II, and III patients accounted for 18 (9.4%), 169 (88%), and 5 (2.6%) individuals, respectively. The mean ICG R15 was 10.3 ± 4.8%. Twenty-seven (14.1%) patients had a previous experience of abdominal surgery before laparoscopic major hepatectomy (Table 2). The mean operative time was 272.2 ± 80.2 min. The mean estimated blood loss was 300.4 ± 252.2 mL and intraoperative transfusion was required in 9 (4.7%) patients. We used the Pringle maneuver for 117 patients (60.9%). The mean time of the Pringle maneuver was 67.3 ± 33.9 min. The mean hospital stay was 9.8 ± 3.6 days and the mean weight of resected specimens was 380.4 ± 201.7 g. Conversion to laparotomy was required in 3 (1.6%) patients. The reasons for conversion were uncontrolled bleeding and bile leakage from the transected Glissonean pedicle in 2 patients and uncontrolled hypercapnea during the operation in 1 patient. Postoperative complications occurred in 6 patients (3.1%). According to the Clavien–Dindo classification, 5 complications were grades I and II; these were resolved by conservative treatment.^[20]^ One patient developed biliary stricture after laparoscopic right hemihepatectomy and required endoscopic retrograde cholangiopancreatography (ERCP) and endoscopic retrograde biliary drainage (ERBD) stent insertion (Table 3). There was no mortality within the first postoperative 90 days.

**Table 1:**
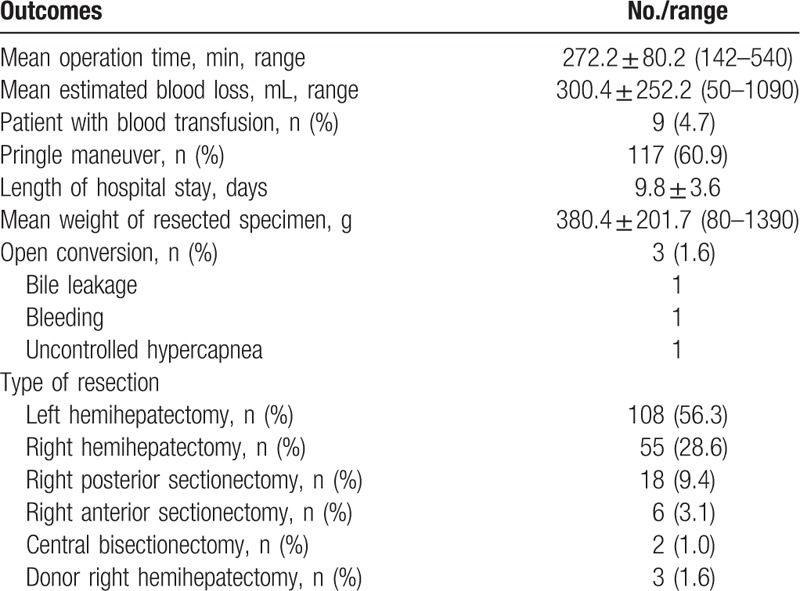
Surgical outcomes and type of resection for patients undergoing laparoscopic major hepatectomy.

**Table 2:**
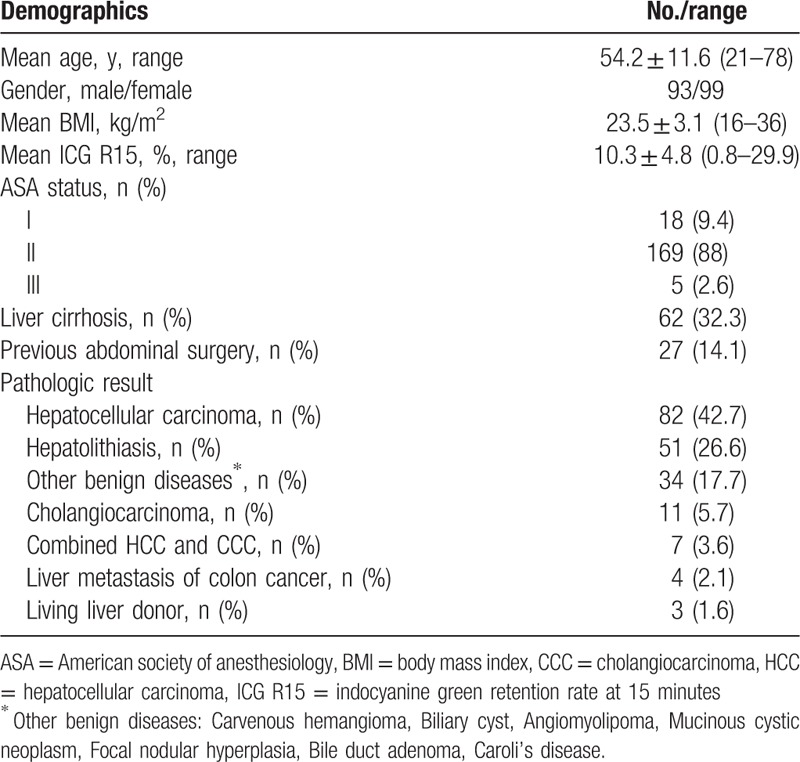
Demographic data and pathologic results of patients undergoing laparoscopic major hepatectomy.

**Table 3:**
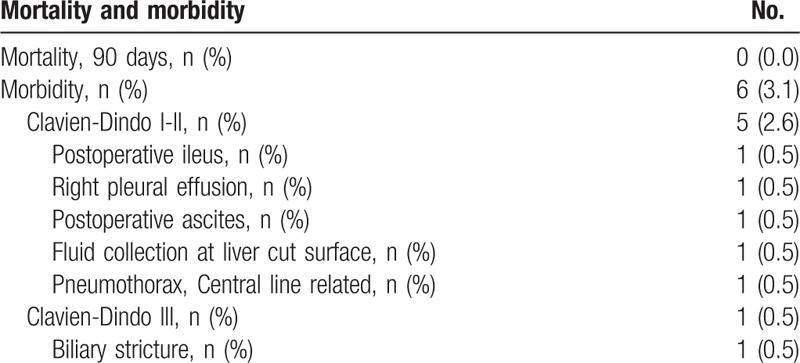
Mortality and morbidity for patients undergoing laparoscopic major hepatectomy.

Postoperative pathology results are shown in Table 2. Pathologically, 104 tumors (54.1%) were malignant lesions (82 hepatocellular carcinomas [HCC], 11 cholangiocarcinomas [CCC], 7 combined HCCs and CCCs, and 4 cases of metastatic colon cancer). Eighty-five (44.3%) cases were benign and most were hepatolithiasis.

**Figure 2:**
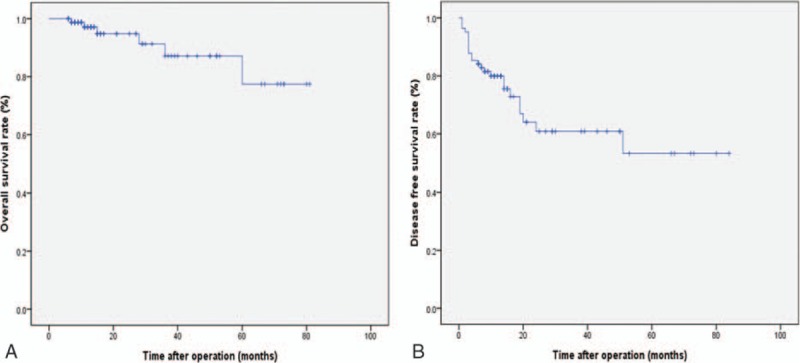
Overall survival (A) and disease-free survival curves (B) for patients with hepatocellular carcinoma.

The clinicopathologic and postoperative outcomes for patients with HCC are summarized in Table 4. Among 82 patients with HCC, 53 (64.6%) displayed liver cirrhosis postoperatively. The most common original disease was hepatitis B virus infection. Fourteen patients were treated by transcatheter arterial chemoembolization (TACE) and 3 patients were treated by radiofrequency ablation (RFA) preoperatively. We performed preoperative portal vein embolization in 2 patients. The mean size and number of resected tumors were 3.2 ± 1.5 cm and 1.04 ± 0.19, respectively. The safe resection margin was, on average, 24.8 ± 20.9 mm. We followed up all the patients with HCC with a mean follow-up period of 24.9 ± 20.9 months. Twenty-four of 82 (29.3%) patients developed tumor recurrence and 6 (7.3%) patients died of tumor recurrence. The disease-free survival rates were 85.4% at 1 year, 79.9% at 3 years, and 64.1% at 5 years. The overall survival rates were 97.1% at 1 year, 87.1% at 3 years, and 77.4% at 5 years (Fig. 2). Demographic data and surgical outcomes of the living donors are shown in Table 5.

**Table 4:**
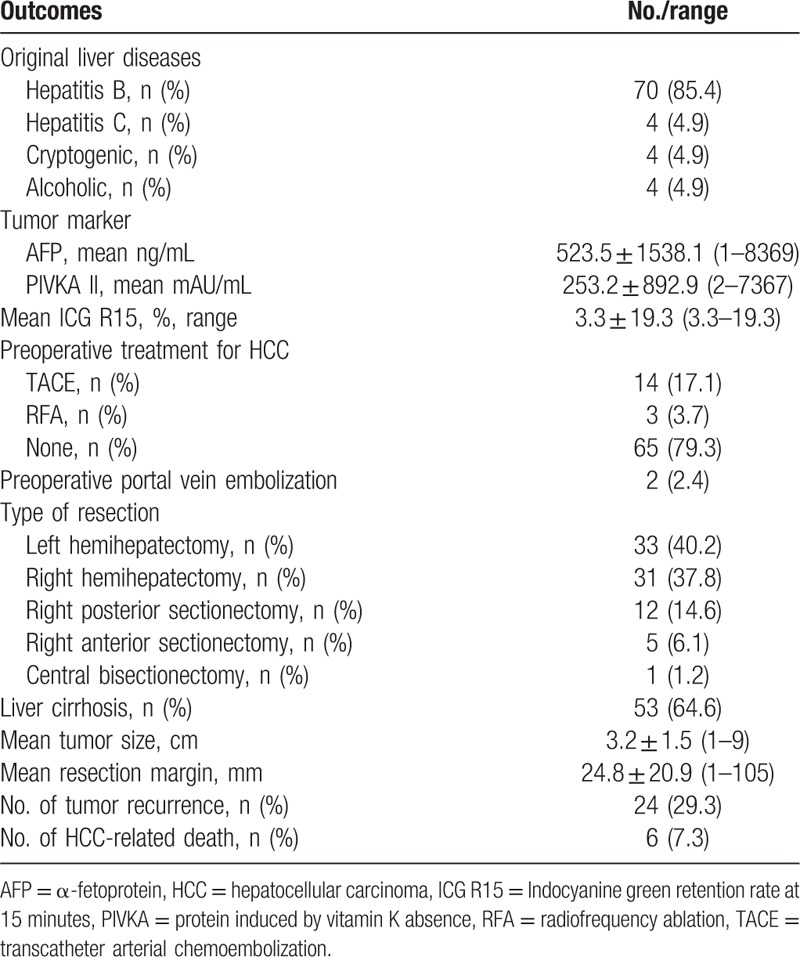
Clinicopathologic and surgical outcomes for patients with hepatocellular carcinoma.

**Table 5:**
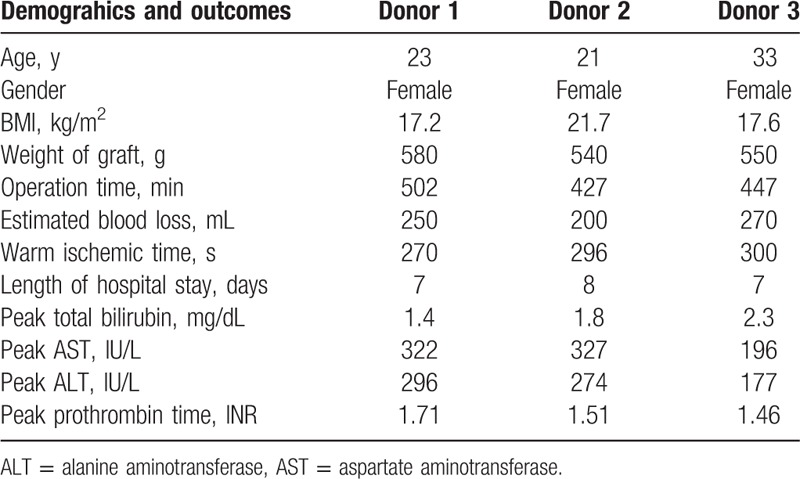
Demographic data and surgical outcomes of the living donors undergoing pure laparoscopic donor right hepatectomy.

## 4 Discussion

Our data provide more evidence of the safety of laparoscopic major liver resection and its feasibility as a surgical option for selected patients. As experience accumulates, laparoscopic major hepatectomy can be the first option for surgery for benign lesions and for cases of malignant tumor and living donors. Even though we did not compare the advantages of the laparoscopic approach with open surgery in this study, our experiences indicate that, compared to open surgery, laparoscopic liver resections produce less pain and reduce the need for analgesic drug use, shorten hospital stay, have fewer transfusion requirements, produce faster recovery, result in less postoperative adhesion, reduce abdominal wall damage and improve cosmetic results, as has been described previously.^[^19 21–26^]^

A systematic review documented complications in 11% to 23% of patients with 3 deaths among 770 patients.^[14]^ In the current study, there was no mortality within the first postoperative 90 days. The morbidity rate was 3.1%. Considering that we have begun to perform laparoscopic major resections only since 2007, our 7-year results are outstanding. Only 1 patient, who had a cholangiocarcinoma adherent to the right main hepatic duct, developed biliary stricture after laparoscopic right hepatectomy. In that case, we had to divide the right hepatic duct just above the bile duct bifurcation to achieve a safe resection margin. We believe that our low mortality and morbidity rates in this study are mainly due to strict patient selection, precise anatomical resection, meticulous operative techniques, and a wealth of experience with open major hepatectomy. Most of our patients were ASA classes I or II, with good general performance. Only patients with Child-Pugh class A cirrhosis were included in this study. Patients with Child-Pugh class B and C cirrhosis were treated with laparoscopic minor hepatectomy, open hepatic resection, nonoperative modalities, or liver transplantation. We checked preoperative CT liver volumetry for all patients. Only patients who met our criteria underwent an operation to prevent posthepatectomy liver failure.

We experienced 3 cases of open conversion (1.6%) because of uncontrolled hypercapnea, bleeding, and bile leakage. The conversion rate was much lower than the ∼10% rate reported in other studies.^[^13 16^]^ Despite the concern about the risk of major bleeding, problematic bleeding occurred in only 1 of 192 cases. Techniques to prevent and to control bleeding are very important in laparoscopic major hepatic resections. We overcame the risk of bleeding using various methods. First, we maintained low central venous pressure during parenchymal transection with the help of an anesthesiologist to reduce backbleeding from the hepatic veins. Second, the additional effect of the pneumoperitoneum explains the reduced amount of bleeding in laparoscopic resections. The most common source of bleeding is parenchymal transection. We used extraparenchymal selective inflow control and meticulous parenchymal transection after identifying the locations of the tumor and the hepatic vein using intraoperative ultrasonography. Also, we transected the parenchyma using a laparoscopic *CUSA*, a meticulous hemostatic instrument.

The extraparenchymal Glissonean approach was preferred for right side hepatectomy and individual dissection was preferred for left hemihepatectomy for inflow control. In left hemihepatectomy, individual isolation and division of the hepatic artery and portal vein was much easier and more convenient than it was when using the Glissonean approach. For the Glissonean pedicle isolation, we used a Goldfinger dissector, which is a very useful instrument. The atraumatic tip and multipositional flexibility are ideal for safe isolation of the Glissonean pedicle. Laparoscopic ultrasound is helpful in identifying the location of a tumor as well as the expected margin of resection. For cases of HCC, the mean resection margin was 24 mm, which is considered acceptable to achieve the oncological goal.

The oncologic outcome after laparoscopic major liver resection is a major issue, and long-term survival data on patients who underwent laparoscopic major liver resection for HCC are lacking. However, several studies have reported that the overall survival rate and disease-free survival rate of laparoscopic liver resection patients are similar to those of individuals undergoing open liver surgery during short-term follow-ups.^[^10 22 27–34^]^ In our study, the 5-year overall survival rate was 77.4% and the disease-free survival rate was 64.1%. However, a randomized prospective controlled clinical trial is necessary to compare the oncologic outcomes of laparoscopic major liver resection and open major liver resection.

The first international consensus is that patients with solitary lesions measuring ≤5 cm located in liver segments II to VI are considered suitable for laparoscopic liver resection.^[19]^ Initially for performing laparoscopic major hepatectomies, our indication was confined to small left-sided tumors measuring ≤5 cm. As our surgical experience accumulated, the surgical indication was extended to right-sided tumors and larger tumors measuring <10 cm. We suggest that tumor size alone is no longer a contraindication to the laparoscopic approach. Rather, tumor location and its relation to other structures, general performance, and underlying liver cirrhosis are more important considerations when choosing the method of operation. Laparoscopic resection for supero-posteriorly located tumors is achievable, though there is a steep learning curve.

We performed 3 cases of donor right hemihepatectomy using the pure laparoscopic approach. Laparoscopic or hand-assisted donor hepatectomy are approved options for donor hepatectomy.^[^35–39^]^ We previously reported the clinical outcomes of laparoscopic donor left lateral sectionectomy in pediatric liver transplant patients^[39]^ and hand-assisted laparoscopic surgery in living donor right hepatectomy.^[40]^ However, pure laparoscopic donor right hemihepatectomy is very risky because of the complexity of liver transection and procurement.^[41]^ Therefore, we confined the donors to those who had a single hepatic artery with a single bile duct and no portal vein variant. There was no donor morbidity and the outcome of transplantation was excellent. Outcomes were comparable to those of liver transplantation using a living donor graft in laparotomy. Considering that most donors are young, the pure laparoscopic approach produced a satisfactory cosmetic outcome, which compared with the significant scar that usually remains after laparotomy. Furthermore, for these cases, the hospital stay was shorter with a more rapid return to daily life than after open surgery.

## 5 Conclusion

Laparoscopic major hepatectomy including pure laparoscopic donor right hemihepatectomy is technically demanding and requires great expertise in both open liver surgery and laparoscopic surgery. However, we believe that laparoscopic major hepatectomy can be a safe and feasible procedure in select patients and donors when performed by an experienced surgeon.

## Acknowledgments

The authors thank Dr. So-Hyun Nam from Dong-A University College of Medicine for statistical consulting and editing this manuscript. The authors have no conflicts of interest or financial ties to disclose.
